# iPSC-Derived Natural Killer Cell Therapies - Expansion and Targeting

**DOI:** 10.3389/fimmu.2022.841107

**Published:** 2022-02-03

**Authors:** Benjamin H. Goldenson, Pooja Hor, Dan S. Kaufman

**Affiliations:** Department of Medicine, Division of Regenerative Medicine, University of California, San Diego, La Jolla, CA, United States

**Keywords:** NK cell, chimeric antigen receptor (CAR), immunotherapy, iPSC (induced pluripotent stem cells), cell engineering

## Abstract

Treatment of cancer with allogeneic natural killer (NK) cell therapies has seen rapid development, especially use against hematologic malignancies. Clinical trials of NK cell-based adoptive transfer to treat relapsed or refractory malignancies have used peripheral blood, umbilical cord blood and pluripotent stem cell-derived NK cells, with each approach undergoing continued clinical development. Improving the potency of these therapies relies on genetic modifications to improve tumor targeting and to enhance expansion and persistence of the NK cells. Induced pluripotent stem cell (iPSC)-derived NK cells allow for routine targeted introduction of genetic modifications and expansion of the resulting NK cells derived from a clonal starting cell population. In this review, we discuss and summarize recent important advances in the development of new iPSC-derived NK cell therapies, with a focus on improved targeting of cancer. We then discuss improvements in methods to expand iPSC-derived NK cells and how persistence of iPSC-NK cells can be enhanced. Finally, we describe how these advances may combine in future NK cell-based therapy products for the treatment of both hematologic malignancies and solid tumors.

## Introduction

Over the past decade, cellular therapies have advanced from pre-clinical studies through clinical trials and now to several U.S. Food and Drug Administration (FDA) approved therapies. Despite these successes, the FDA approved chimeric antigen receptor (CAR) T cell therapies for B-cell acute lymphoblastic leukemia (B-ALL), B-cell lymphomas and multiple myeloma are limited by their manufacturing processes and treatment-related toxicity (1).

Some of the major challenges with autologous CAR-T cell therapy are antigen escape, limited capability of CAR-T cells to migrate to and infiltrate the immunosuppressive tumor microenvironment (TME), and treatment-associated toxicities. The most significant adverse effects of CAR-T cells are cytokine-release syndrome (CRS) and immune effector cell-associated neurotoxicity syndrome (ICANS) (2). CRS occurs in approximately 25% of patients treated with anti-CD19 CAR-T cells, severe ICANS in 12-42% and non-relapse related death in 1-2% of treated patients (3–6). An additional manufacturing issue with autologous CAR-T cell therapies is that patients who have previously received multiple rounds of chemotherapy may not be able to mobilize sufficient T cells for CAR-T cell production with up to 10-30% of patients that fail CAR-T cell manufacturing (3, 5, 7). Additionally, in the time required for CAR-T cell manufacturing, patients can experience disease progression. For example, 38 out of 165 patients enrolled in one study of anti-CD19 CAR-T cells dropped out before receiving therapy (5). Therefore, 30% or more of patients who could potentially benefit from CAR-T cell therapy do not receive the treatment. Additionally, the cost of CAR-T cell manufacturing is typically $300,000-$500,000 for each patient, even before the costs of care.

Due to these limitations of autologous CAR-T cells, allogenic T cell approaches and alternative cell sources for cellular therapy have been investigated. Collecting allogenic, healthy and functional T cells from volunteer donors instead of the cancer patient undergoing chemotherapy has been one approach; however, allogenic T cells must be modified to prevent the development of graft versus host disease (GVHD) (8). Allogeneic T cells have been engineered to delete their human leukocyte antigen (HLA) class I and II molecules and disrupt T-cell receptor (TCR) expression to evade immune rejection and reduce GVHD in patients (7, 9–12).

Natural killer (NK) cells, key effector cells of the innate immune system, possess features that can overcome many of the challenges associated with autologous CAR-T cells. NK cells are an ideal cell population for anti-cancer cell therapy as the repertoire of receptors that regulate NK cell activity are distinct from the TCR system and allow for use of NK cells as an allogeneic therapy (13, 14). Therefore, NK cells do not require HLA matching and multiple clinical studies demonstrate a lack of GVHD despite these being allogeneic cells, making them a relatively safer therapeutic approach compared to allogeneic CAR-T cells that can still lead to GVHD if there are any cells with residual TCR (15–19). NK cells are known to play a key role in immunosurveillance that can limit or prevent tumorigenesis (20). This ability for NK cells to provide natural immunity to malignancies has been demonstrated in both mice and humans (21, 22). Agents that enhance endogenous NK cell activity can lead to improved anti-tumor responses (13). For example, the anti-NKG2A monoclonal antibody Monalizumab that blocks this inhibitory receptor expressed on NK cells and cytotoxic T cells has demonstrated potent anti-tumor activity in clinical trials (23, 24). NK cells are also recognized to play a key role in the anti-tumor activity of allogeneic hematopoietic cell transplantation (25). Because of these potential advantages, NK cells obtained from various sources have been tested as a specific cell population for adoptive transfer to treat cancer patients in clinical trials. These sources include the NK-92 cell line, peripheral blood cells, umbilical cord blood (CB), and induced pluripotent stem cells (iPSCs) (16, 26–28). iPSC-derived NK cells provide added benefits in terms of relative ease of genetically engineering, clonal selection post-genetic modification and no requirement for cells to be collected from a donor at any point in time. However, the scale-up and manufacturing of NK cells starting from iPSCs can be more challenging, though has been routinely accomplished (29).

## NK Cells as Cellular Therapy

Distinct NK cell sources each possess advantages and disadvantages for use in cellular therapy targeting cancer (30). Peripheral blood NK (PB-NK) cells must be collected from a donor by apheresis and expanded prior to use (16, 31, 32). CB-NK cells are required to be obtained from an umbilical cord blood unit and expanded (28, 33, 34). CB-NK cell populations can be expanded and exhibit similar cytotoxicity to PB-NK cells against tumor cells post expansion (34, 35). For both PB- and CB-NK cells there is variability in the NK cell yield from each blood unit which is influenced by donor variability and dependent on NK cell yield post-purification (15, 36). NK cell lines such as NK-92 provide homogeneous cell populations that expand indefinitely in culture and are more amenable to genetic alteration (26). However, these cell lines lack important receptors typically expressed on NK cells. For example, NK-92 cells do not express Killer Ig-like receptors (KIRs) or CD16, an Fc receptor that plays an important role in activating antibody-dependent cellular cytotoxicity (ADCC) (26). Additionally, NK tumor cell lines such as NK-92 cells are aneuploid and for safety reasons must be irradiated prior to patient administration. This irradiation limits their ability to expand and persist *in vivo*, decreasing anti-tumor efficacy (26). Pre-clinical studies and clinical trials of cellular therapies have demonstrated that improved CAR-T cell persistence corresponds with better treatment efficacy (37, 38). Similar studies for NK cells have also shown that persistence in pre-clinical *in vivo* models correlates with better tumor killing (39, 40). Therefore, this limited expansion and persistence after being administered to patients may account for the limited efficacy of NK-92 cells in several clinical trials (41, 42).

Multiple clinical trials using these different NK cell products demonstrate the efficacy of allogeneic NK cell adoptive transfer therapy. The ability of unmodified allogeneic NK cells to kill tumors that are resistant or refractory to standard therapies has been most clearly demonstrated in the treatment of acute myeloid leukemia (AML) (16, 17, 43–45). The first, seminal study using PB-NK cells was done by Miller et al. who treated patients with relapsed/refractory AML with allogeneic PB-NK cells from haploidentical donors. Complete hematologic remission was obtained in five of nineteen patients (16). In a larger study of 42 patients with AML treated with haploidentical NK cells and IL-15 by the same group, approximately 40% of patients achieved complete remission (45). A separate study of AML patients treated with haploidentical NK cells combined with an immunotoxin to deplete IL2 receptor-expressing T-regulatory cells led to 53% compete response rate (44).

Romee, Fehniger and colleagues demonstrated that stimulation with the cytokines interleukin-12 (IL-12), IL-15, and IL-18 produces so-called cytokine-induced memory-like (CIML) NK cells that exhibited a 56% overall response rate and 44% complete response rate in treatment of acute myeloid leukemia (46–48). Another phase 1 clinical trial by Green Cross LabCell Corporation used allogeneic NK cells (named MG4101) derived from peripheral blood in combination with rituximab for patients with B cell lymphomas (49, 50). No patients experienced dose-limiting toxicities and five out of nine patients experienced a response.

Additional trials using NK cells engineered to improve targeting of tumors and NK cell expansion have been initiated (15, 51–53). For example, a recent trial utilizing adoptive transfer of *ex vivo* expanded, HLA-mismatched, CB-NK cells engineered to express both an anti-CD19 CAR and secreted IL-15 were used to treat 11 patients with CD19-positive relapsed or refractory B cell malignancies and demonstrated objective response in 73% of the patients (15). Importantly, none of the patients developed serious toxicities associated with CAR-T cells including cytokine release syndrome, neurotoxicity, and GVHD (15).

## iPSC-NK Cells – A Standardized, Off-the-Shelf Alternative for Cellular Therapy

NK cells generated from pluripotent stem cells have also emerged as a promising strategy to produce standardized, off-the-shelf NK cells with improved anti-tumor activity. This approach circumvents many of the challenges seen with other NK cell populations and T cells for adoptive cell therapy, such as the requirement for collection from a donor or cord blood unit. In contrast, pluripotent stem cells, either human embryonic stem cells (hESCs) or iPSCs, can grow indefinitely in an undifferentiated state *via* self-renewal (54–56). Therefore, the ability to routinely derive NK cells from hESCs and iPSCs allows for an unlimited number of uniform NK cells to be produced from the starting pluripotent stem cell population to provide a standardized, off-the-shelf approach.

The use of hESCs or iPSCs to derive engineered cell products also enables individual clone isolation and detection of off-target genomic alterations *via* whole-genome sequencing (39, 57). This approach also allows for the efficient addition of multiple genetic alterations to augment NK cell cytotoxicity. Genetic engineering approaches such as transposons and lentiviral delivery ensure efficient transgene insertion and stable expression in iPSCs (58, 59). TALENS and CRISPR/Cas9 can also be used for more precision in knocking in or deleting specific genes (60–64). Once engineered, the engineered and undifferentiated iPSCs can be frozen and stored to allow for consistent production of NK cells with an identical phenotype.

The first studies of human pluripotent stem cells demonstrated that hESCs can be differentiated into the three primary germ layers (54). Further studies led to the differentiation of CD34^+^ hematopoietic progenitor cells and specific myeloid, erythroid, and lymphoid lineage populations (65–70). With the advent of iPSC technology, laboratories worldwide have developed protocols to differentiate target cells of many lineages with hopes of use for cellular therapy for complex diseases. Improvement in methods to derive NK cells from hESCs/iPSCs now enables the production of homogeneous, functional NK cells at a clinical scale (29). Initial methods to derive cells of hematopoietic origin involved coculturing of hESCs with irradiated stromal cell lines to generate CD34^+^CD45^+^ hematopoietic progenitors (29, 71). This was followed by use of a second stromal cell line combined with defined cytokines to produce mature NK cells (29, 71). Subsequent studies refined NK cell production from hESCs/iPSCs to eliminate the use of serum-containing media and stromal cells. A “spin embryoid body (EB)” protocol produces hematopoietic organoids that contain hematopoietic progenitor cells, as well as endothelial and mesenchymal cells. These hematopoietic progenitor cells then differentiate into NK cells under defined conditions (72, 73). The hESC/iPSC-derived NK cells can also be further expanded in the presence of IL-2 and K562 cells engineered to present 4-1BB ligand and IL-21 to the NK cells (29, 31). hESC/iPSC-derived NK cells recapitulate many key features of primary NK cells. They express important NK cell markers such as CD56, CD94, NKG2D, NKp44, NKp46, CD16, and KIRs, and exhibit potent cytotoxicity toward diverse solid tumors and hematological malignancies (69, 74, 75). Other methods to derive NK cells from human iPSCs have also been demonstrated, including developmental and functional differences between NK cells derived under Wnt-dependent versus Wnt-independent conditions (76).

Like CB- and PB-NK cells, hESC/iPSC-derived NK cells exhibit cytotoxicity against diverse target cells *via* lytic granule release of perforins and granzymes, production of proinflammatory cytokines interferon gamma (IFN-γ) and tumor necrosis factor alpha (TNFα), and direct cell contact mediated apoptosis through TRAIL and Fas-FasL interaction (57, 77). However, NK cells derived from iPSCs are equally or more effective as primary NK cells and NK cell lines. *In vivo* ovarian cancer xenograft models demonstrated iPSC-NK cytotoxicity was comparable to PB-NK cells (27). A different group found that iPSC-NK cells have greater cytotoxicity against multiple ovarian, colon and breast cancer cell lines compared to donor PB-NK cells (78).

## Improvement of iPSC-NK Cell Expansion and Function Through Genetic Engineering

**Figure 1:**
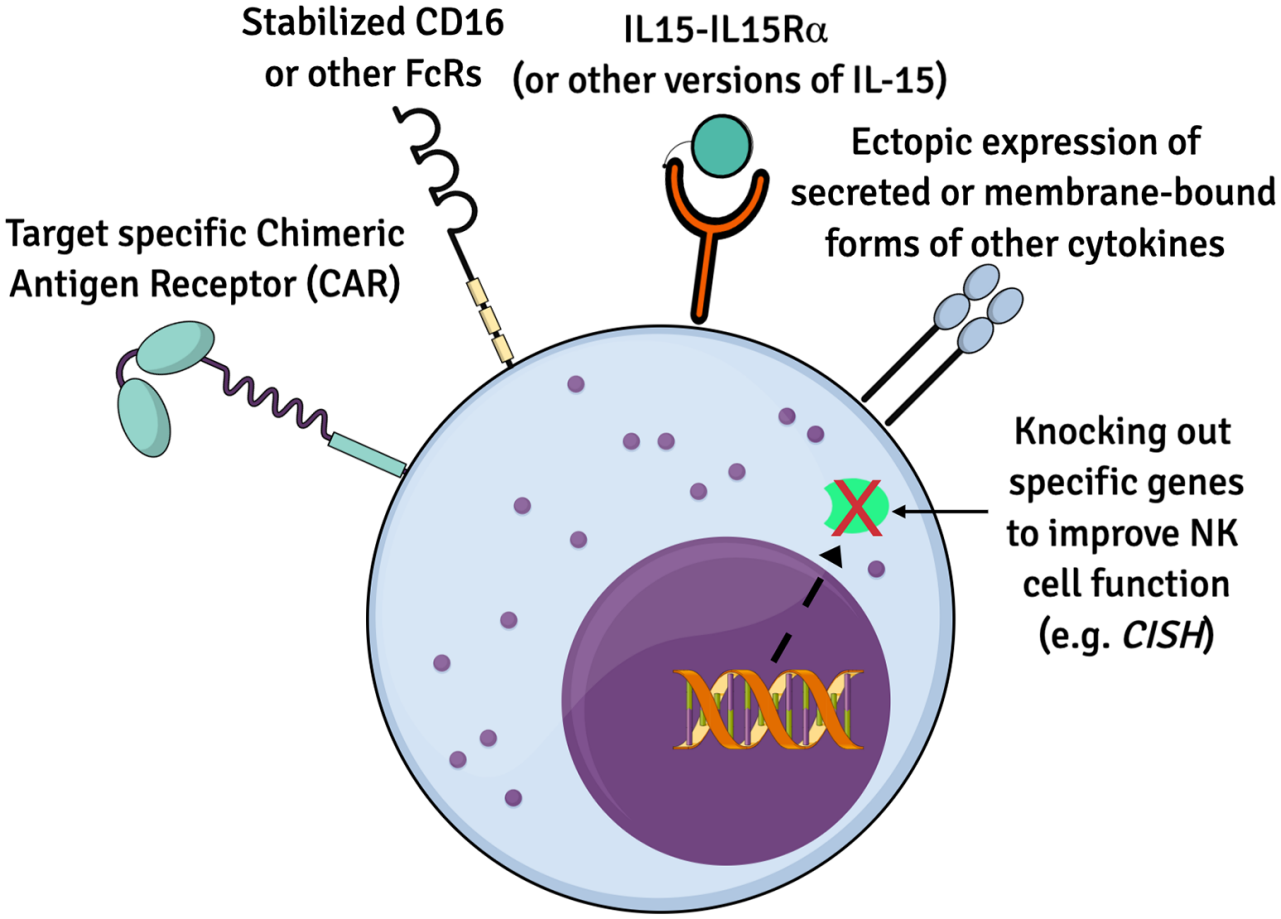
Summary of genetic modifications to improve iPSC-NK cells. Numerous genetic alternations have been engineered to enhance the biology and function of iPSC-derived NK cells for therapeutics. Ectopic expression of IL15 and/or other cytokines, CARs to boost anti-tumor cytotoxicity, recombinant CD16 and knockout of specific genes such as *CISH* are some of the approaches.

Multiple recent studies have genetically engineered iPSCs to create iPSC-NK cells with enhanced expansion, *in vivo* persistence and tumor killing capability are being explored (52, 79, 80). Many of these technologies were first developed and tested in PB-NK cells and/or CB-NK cells and subsequently translated into iPSC-derived NK cells. The iPSCs provide a stable platform for routine genetic modifications that only need to be done on a one-time basis. Once a stably engineered iPSC clone is identified, this can be expanded and used to produce a standardized population of appropriately engineered iPSC-derived NK cells. Some examples of strategies to enhance NK cell functions are described in this section (**Figure 1**).

### Improving NK Cell Anti-Tumorigenic Activity and Expansion *via* IL-15 Pathway

IL-15 plays an important role to stimulate NK cell expansion and cytotoxic functions (13, 81–84). IL-15 activation has also been shown to mitigate the immunosuppression mediated by transforming growth factor (TGF)-β1, released from the TME (85). These traits have made manipulation of IL-15 expression an appealing strategy to enhance the anti-tumor activity of a variety NK cells populations without the need to supplement the cell production cultures with high doses of cytokines. Various methods to activate the IL-15 signaling pathway have shown to improve NK cell biology and function. The Rezvani group demonstrated that expression of IL-15 combined with an anti-CD19 CAR improved CB-derived NK cell cytotoxicity towards CD19-expressing cell lines and primary leukemia cells *in vitro*, and markedly extended survival in a Raji lymphoma xenograft model (86). This approach was translated into a clinical trial of anti-CD19 CAR-expressing CB-derived NK cells that were well tolerated and demonstrated a 73% overall response rate for patients with relapsed/refractory B cell malignancies (15). Another study by Imamura et al. demonstrated expression of a membrane bound form of IL-15 (mbIL-15) in human PB-NK cells enhanced anti-tumor killing against hematologic malignancies and solid tumors by augmenting NK cell survival and expansion *in vitro and in vivo* without the need of additional exogenous cytokines (87). Another approach employed by two groups used an IL-15 receptor fusion construct comprising of an IL-15 superagonist and IL-15 receptor α (IL-15SA/IL-15RA) to increase anti-tumor activity of PB-NK and iPSC-NK cells, respectively, *in vitro* and *in vivo* (40, 88).

Regulators of IL-15 signaling also provide a target to improve NK cell function. Cytokine-inducible Src homology 2–containing (CIS) protein, encoded by the *CISH* gene, is a key negative regulator of IL-15 signaling. Initial studies demonstrated that deletion of *CISH* in mice leads to increased sensitivity to IL-15, enhanced metabolism and improved anti-tumor activity of NK cells (89, 90). The findings were adapted to human iPSC-NK differentiation platform by using CRISPR/Cas9 edited *CISH*-knockout (CISH^−/−^) iPSCs and differentiating them into CISH^−/−^ iPSC-NK cells which demonstrate improved metabolic profile, *in vivo* persistence and increased anti-tumor activity through increased IL-15-mediated JAK-STAT signaling activity (39, 91). Similar work also demonstrates that deletion of *CISH* in PB-NK cells or UCB-NK cells can also improve their anti-tumor activity (89, 91, 92).

### Effects of Other Cytokines and Chemokines on NK Cell Expansion, Metabolic Fitness and *In Vivo* Persistence

NK cell activity is regulated by interactions with diverse immune cells including, but not limited to, T cells, dendritic cells, macrophages, and bone marrow stromal cells. These cells secrete diverse cytokines and chemokines that bind to specific receptors on NK cells. These cytokine receptors include IL-2R, IL-12R, IL-15R, IL-18R, IL-21R (93, 94).

Treatment of NK cells with cytokines allows NK cells to acquire an increased spectrum of effector functions (95). IL-18 is a key player of this priming process. Studies found that NK cells isolated from IL-18-KO mice secreted significantly less IFN-γ than wild-type NK cells in response to stimulation with IL-12 or IL-2 plus IL-12, demonstrating cooperation between the IL-2 and IL-18 signaling pathways (96). Another recent study demonstrated increased *ex vivo* expansion and cytotoxic activity of treated with a cytokine cocktail of IL-2, IL-15, IL-18 (97).

In pre-clinical and clinical studies, the Fehniger group has shown that treatment with a cytokine cocktail consisting of IL-12, IL-15 and IL-18 results in development of CIML NK cells with enhanced interferon-γ (IFN-γ) production and cytotoxicity against leukemia cell lines or primary human AML blasts (46–48). Their phase I clinical trial resulted in 4 out of 9 patients achieving complete remissions (48). CIML NK cells further demonstrated heightened cytotoxicity, enhanced IFN-γ production and persistence against ovarian cancer and other malignancies (98, 99).

IL-21 is another common γ-chain cytokine crucial for NK cell maturation and proliferation (100). In an interesting study, Li et al. demonstrated while increasing concentration of IL-21 (1-10 ng/ml) resulted in higher cytotoxicity through upregulation of IFN-γ and granzyme B, at high concentrations (50 ng/ml) IL-21 resulted in NK cell apoptosis (101). Notably, several groups now routinely utilize irradiated NK cell-sensitive tumor cells that express membrane-bound IL-21 (mbIL-21) and other stimulatory ligands (e.g., 4-1BBL or Ox40L) to stimulate prolonged and large-scale expansion of NK cells (29, 31, 102–104).

Efficient tumor infiltration and homing of NK cells is vital for effective anti-tumor activity. However, cells within the TME secrete chemokines such as C–X–C motif chemokine ligand 8 (CXCL8) or C–C motif chemokine ligand 2 (CCL2) that suppress the activity of intratumoral NK cells (105). High concentrations of adenosine in primary and metastatic TME, specifically myeloid cell adenosine A2A receptors (A2ARs) have a myelosuppressive effect that leads to suppression of NK cell anti-tumor activity (106). Additionally, IL18 binding protein (IL18BP) is a decoy receptor found in the TME that binds to IL-18 with high affinity (107). IL18BP reduces the efficacy of endogenous IL-18 or recombinant IL-18 (rIL-18) administered to try to mediate improved anti-tumor activity (108). Patients treated with rIL-18 have 10- to 100-fold higher concentrations of IL-18BP in their serum (107, 109, 110). In a fascinating recent study, IL-18 was engineered to override the IL18-BP inhibition *via* a ‘decoy-resistant’ IL-18 (DR-18) that was able to stimulate NK cells to effectively treat PD-1 resistant tumors despite the presence of IL18BP (111).

A detailed transcriptomic analysis demonstrated *ex vivo* expanded NK cells had drastic differences in expression pattern of chemotactic receptors and ligands, including a significant downregulation of CXCR4 and consequent upregulation of CCR5. The study further observed knocking out CCR5 resulted in reduced NK cell trafficking into liver and corresponding increase in NK cell presence in the blood circulation in immunodeficient mice post-infusion (105). PB-NK cells transfected with CCR7 had increased towards CCL19, a lymph node-associated chemokine (112). CXCR2-expressing primary NK cells also showed improved migration to renal cell carcinoma (113). Dual expression of an anti-EGFRvIII CAR and CXCR4 led to increased anti-tumor and better survival in xenograft mouse models (114).

### Effect of Hypoxia on NK Cell Function

The hypoxic TME is a characteristic feature of solid tumors. Hypoxia-inducible factors (HIFs) are activated at low oxygen (115–118). Notably, deletion of HIF-1α in mouse NK cells inhibits tumor growth despite reducing cytolytic activity of NK cells. This was mainly shown to be mediated *via* increased bioavailability of the major angiogenic cytokine vascular endothelial growth factor (VEGF) (119). However, in a recent single cell transcriptomic analysis, conditional deletion of HIF-1α in mouse tumor-infiltrating NK cells lead to increased NK cell activation, upregulated NF-kB signaling and improved anti-tumor activity (120).

## Strategies to Improve Tumor Targeting of NK Cells

In addition to strategies to improve function of NK cells, diverse methods have now been used to improve NK cell targeting against more NK cell-resistant tumors. This section describes some of these strategies that include addition of CARs to NK cells, modification of Fc receptors on NK cells, use of immune checkpoint inhibitor antibodies and NK cell engager molecules.

### Development of CAR-Expressing NK Cells

CARs are engineered cell surface receptor constructs that direct immune cell function *via* recognition of the target antigen on the tumor cell surface leading to activation of the immune effector cell *via* an intracellular signaling domain (121–123). CAR constructs typically contain three main components: an ectodomain for recognition of the target antigen (the binder), a transmembrane domain (TM) and an intracellular signaling endodomain(s) (124–126). The ectodomain is typically an immunoglobulin-like single-chain variable fragment (scFv) that imparts antigen specificity against the target tumor. For example, scFvs that target CD19 to treat B cell leukemia and lymphoma are now used for the FDA-approved CAR-T cells (3, 5). Binders that target mesothelin, epidermal growth factor receptor, prostate specific membrane antigen or other tumor antigens have been developed and are in clinical trials to treat diverse malignancies (51, 127–129).

NK cell CAR-based therapy has been shown to benefit from utilization of NK cell-specific CAR constructs compared to CARs that were developed for T cells. For example, our group tested four different transmembrane domains (CD16, NKp44, NKp46, and NKG2D) and four different costimulatory domains (2B4, DAP10, DAP12, and CD137) in combinations with CD3ζ to optimize an NK cell-specific CAR construct. These studies demonstrated a CAR that contains the NKG2D transmembrane domain and 2B4 co-stimulatory domain mediated improved anti-tumor activity both *in vitro* and *in vivo* (128). Other groups have engineered NK cells to express CARs targeting CD19, CD33 or GPC3 using the 4-1-BB and CD3ζ components to kill otherwise resistant tumor cells (53, 130, 131). Additional studies have used NK cells that express CARs that incorporate either DNAX-activation protein 10 or 12 (DAP10 or DAP12) as the activating domain or as a costimulatory domain alongside CD3ζ (114, 128). A CAR consisting of NKG2D-DAP10-CD3ζ domains increased NK cell-mediated cytotoxicity and cytokine secretion against leukemia and solid tumor cell lines (132). A DAP12 signaling domain expressed in NK cells outperformed CD3ζ expression alone in first-generation prostate stem cell antigen targeting CAR-NK cells (133).

### Increased CD16 and CD64 Expression Enhances ADCC Mediated by iPSC-NK Cells

NK cells express the activating immunoglobulin gamma Fc receptor CD16a which recognizes the Fc region of IgG antibodies bound to target targets. CD16a engagement provides a potent stimulus to activate NK cells (134). The clinical anti-tumor activity of monoclonal antibody therapy is in-part dependent on this NK cell ADCC activity (135). For example, there are allelic variants of CD16a with different Fc binding affinities, and the high affinity CD16 variant (F158V) has been shown to lead to improved antitumor responses in patients treated with monoclonal antibodies (136, 137). Additionally, as a negative feedback mechanism, CD16a is cleaved from the surface of activated NK cells by the metalloprotease ADAM17, resulting in decreased CD16a expression and decreased ADCC. With genetic modification, the ADAM17 cleavage site on CD16a can be mutated to block CD16a shedding and increase ADCC (82, 138). In iPSC-NK cells, a CD16 molecule with the high affinity F158V mutation that is resistant to ADAM17 cleavage (termed hnCD16) maintained CD16a surface expression and demonstrated increased cytotoxicity and cytokine production in combination with anticancer monoclonal antibodies (57). *In vivo* efficacy was confirmed in a xenograft mouse model of B cell lymphoma, where anti-CD20 rituximab monoclonal antibodies in combination with hnCD16-iPSC-NK cells improved survival over the combination of PB-NK cells with rituximab or WT iPSC-NK cells.

A second Fc receptor, CD64, binds to the same IgG1 and IgG3 isotypes as CD16A with more than 30-fold higher affinity. However, CD64 is typically only expressed on myeloid cells and not on NK cells (139). Expression of a recombinant receptor consisting of the extracellular region of CD64 and the transmembrane and intracellular regions of CD16a was tested in iPSC-NK cells to determine if this higher affinity Fc receptor could cytotoxicity against tumor cells in combination with monoclonal antibody treatment (140). iPSC-NK cells expressing the CD64/16A chimeric receptor killed EGFR^+^/HER2^+^ SKOV3 ovarian cancer cells when combined with the anti-HER2 therapeutic mAb trastuzumab, or the anti-EGFR1 monoclonal antibody cetuximab, while little anti-tumor activity killing was seen without addition of these antibodies (140). Additionally, the higher affinity of CD64 allowed for monoclonal antibodies to be pre-adsorbed to the NK cells expressing the recombinant CD64 and improved tumor targeting without additional antibody use (140).

### NK Cells Enhance Anti-Tumor Activity in Combination With Immune Checkpoint Inhibitors

Immune checkpoint inhibitor therapies such as anti-programmed death 1 (PD-1) and anti-cytotoxic T-lymphocyte-associated protein 4 (CTLA-4) monoclonal antibodies, that block inhibitory signals on immune effectors cells thereby activating the immune system, have revolutionized oncology (141, 142). The combination of cellular therapy with immune checkpoint inhibition can mediate improved anti-tumor activity. For example, the ability of adoptive transfer of NK cells to augment checkpoint inhibition therapies has been investigated in hematologic and solid tumors models (143–145). iPSC-NK cells combined with PD-1 checkpoint blockade produced more inflammatory cytokines and exerted increased cytotoxicity. In these studies, iPSC-NK cells were shown to cooperate with T cells to enhance inflammatory cytokine production and tumor killing (143). Other NK cell immune checkpoints, such as the inhibitory receptor NKG2A can be blocked to improve anti-tumor activity. The humanized anti-NKG2A antibody Monalizumab was shown to increase NK cell activation, increase tumor killing, decrease tumor volume and increase survival *in vivo* (146). This effect was augmented by simultaneous PD-1 inhibition and now is under study in phase II clinical trials (146).

### Engager Molecules Direct iPSC-NK Cells to Target AML

Following the clinical success of bispecific engagers such as blinatumomab, a CD3-CD19 bispecific antibody that engages CD3+ T cells and traffic them to CD19^+^ B cell acute lymphoblastic leukemia, several groups have developed multi-valent targeting molecules that specifically engage NK cells in close proximity to the target tumor to improve tumor killing (147). These bispecific killer engagers (BiKEs) or trispecific killer engagers (TriKEs) have been designed to stimulate NK cell activating cell surface cell receptors. For example, engagers targeting NK cells to CD30^+^ lymphomas, CD33+ myelodysplastic syndrome, CD133^+^ colon cancer, CLEC12A^+^ and CD33^+^ AML are all in clinical development. A bispecific CD30xCD16 engager was able to direct PB-NK and CB-NK cells to increase cytotoxicity against CD30^+^ lymphomas in a pre-clinical study both *in vitro* and *in vivo* (148). A CD16xCD33 bispecific engager and TriKE targeting CD16, CD33 and stimulating IL15 improved NK cell killing of CD33^+^ myelodysplastic syndrome cells (149, 150). NK cells were directed to more effectively kill CD133^+^ or EPCAM^+^ colon cancer cells by CD16xCD133 or CD16xEpcam TriKEs that included an IL-15 crosslinker (151, 152). In AML preclinical models BiKEs and TriKEs targeting CD33 and CLEC2A on AML increased NK cell mediated killing of CD33^+^ or CLEC2A^+^ AML cells, respectively (153, 154).

NKG2C is another NK cell surface receptor that delivers a strong activating signal to NK cells. To determine if NKG2C signaling could enhance NK cell-mediated antitumor responses an anti-NKG2C/IL-15 engager was developed. The engager has multiple functions, it is designed to bind CD16 to target the NK cells to CD33 that is expressed highly on AML cells, as well as to activate IL-15 signaling and NKG2C. The engager was demonstrated to direct NKG2C^+^ iPSC NK cells to target CD33^+^ AML cells and induce degranulation, IFN-γ production and cytotoxicity against the CD33^+^ cells and primary AML blasts (155).

These strategies to enhance NK cell function can also be combined. Again, iPSCs become very useful for these combined approaches, as it is possible to do all the engineering steps in the undifferentiated iPSCs. Once stable iPSCs are obtained, they can be characterized for any off-target effects of the genetic modification to help ensure safety and uniformity of the differentiated product. The stably engineered iPSCs can then be differentiated into NK cells and expanded for clinical use. This approach was recently described for a product with expression the non-cleavable, high-affinity version of CD16 to allow improved ADCC combined with an IL15-receptor fusion protein to enhance expansion of the cells (40). Additionally, as these NK cells are intended to be combined with an anti-CD38 antibody (Daratumumab) to target multiple myeloma, CD38 was deleted from the iPSCs to produce CD38-knockout (KO) iPSC-NK cells that also contain the engineered CD16 and IL15 molecules. Since CD38 also mediated NAD metabolism, these CD38-KO iPSC-NK cells have features similar to so-called adaptive NK cells that arise after cytomegalovirus infection (40). Interestingly, while these triple-engineered iPSC-NK cells demonstrate potent anti-tumor activity *in vitro*, they were no better than iPSC-NK cells with just the engineered CD16 and IL15 receptor (and not the CD38-KO) in killing tumor cells *in vivo* using myeloma and AML xenograft models (40). Clinical trials utilizing these engineered iPSC-NK cells are underway.

## Conclusion

CAR-T cells have produced impressive clinical results in patients with relapsed or refractory B-cell malignancies and multiple myeloma, with ongoing studies in progress against many other tumor types (1–3, 5). However, the current CAR-T therapeutic strategy has several safety and logistical limitations that reinforce the need to identify alternative immune cell populations for use for cellular therapy. NK cells, and particularly iPSC-NK cells, are a promising alternative to T cells for cellular therapy based their proven safety record, ability to be used as an allogeneic treatment, and ability to be produced in large numbers and be stored to make an off-the-shelf therapy. Questions about NK cell persistence, the durability of the response, homing to the target tumor and the ability to overcome immune checkpoints remain to be answered. Advances in iPSC-derived NK cell expansion and targeting *via* genetic engineering and gene-editing techniques promise to solve many of these issues and move iPSC-derived NK closer to being an approved clinical option for the treatment of hematologic and solid malignancies.

## Author Contributions

BG and PH contributed equally to this work. BG and PH wrote the manuscript. DK reviewed and edited the manuscript. All authors read and approved the submitted manuscript.

## Funding

Studies in the Kaufman lab related to this work are supported by NIH/NCI (U01CA217885) and the California Institute of Regenerative Medicine (TRAN1-10587) and the Sanford Consortium for Regenerative Medicine.

## Conflict of Interest

DK is a co-founder, board member and advisor to Shoreline Biosciences and has an equity interest in the company. DK also consults for Qihan Biotech and VisiCELL Medical for which he receives income and/or equity. DK also has patents in the area of iPSC-NK cells. The terms of these arrangements have been reviewed and approved by the University of California, San Diego in accordance with its conflict-of-interest policies.

The remaining authors declare that the research was conducted in the absence of any commercial or financial relationships that could be construed as a potential conflict of interest.

## Publisher’s Note

All claims expressed in this article are solely those of the authors and do not necessarily represent those of their affiliated organizations, or those of the publisher, the editors and the reviewers. Any product that may be evaluated in this article, or claim that may be made by its manufacturer, is not guaranteed or endorsed by the publisher.
